# Osteomyelitis of the Hip due to *Granulicatella adiacens*: A Case Report of an Extremely Rare Cause of Bone Infection

**DOI:** 10.1155/crdi/7967434

**Published:** 2026-07-22

**Authors:** Niccolò Garofalo, Lorenzo Onorato, Margherita Macera, Ilaria De Luca, Gabriele Martin, Gianluca Conza, Annalisa Itro, Nicola Coppola, Giuseppe Toro

**Affiliations:** ^1^ Department of Medical and Surgical Specialties and Dentistry, University of Campania ‘Luigi Vanvitelli’, Via L. De Crecchio 4, Naples 80138, Italy; ^2^ Department of Mental Health and Public Medicine, Section of Infectious Diseases, University of Campania Luigi Vanvitelli, Via L. Armanni 5, Naples 80131, Italy, unina2.it

**Keywords:** antibiotic, case report, *Granulicatella adiacens*, hip arthritis, multistage, osteomyelitis, treatment

## Abstract

**Background:**

*Granulicatella adiacens (G. adiacens)* is a nutritionally variant *Streptococcus* generally observed in the human mouth microbiota. Although *G*. *adiacens* is involved in bacterial endocarditis and bacteremia, bone and joint infections are extremely rare. In the present study, we reported the first known case of hip osteomyelitis related to *G. adiacens*. Moreover, a commentary on the available evidence was also performed.

**Case Presentation:**

A 59‐year‐old male with a former substance use disorder who initially complained of low back pain with a medical history negative for any other comorbidities, albeit absence seizures. After an accurate evaluation, hip osteomyelitis caused by *G. adiacens* was diagnosed. In our patient, neither bacteremia nor infectious endocarditis was reported, as well as previous oral surgery. The diagnosis of *G. adiacens* was performed through 16S rRNA gene sequencing. A three‐stage surgery was performed based on surgical debridement and antibiotic‐cemented spacer implant, antibiotic‐cemented spacer renewal, spacer removal, and prosthesis implantation. Throughout the entire period, the patient received antibiotic therapy with doxycycline and cotrimoxazole. At the final follow‐up, the patient was infection‐free, but a poor hip function was reported.

**Conclusion:**

*G. adiacens* is rarely associated with bone osteomyelitis, probably because of the difficulty in culture isolation that might lead to misdiagnosis, especially in cases of polymicrobial infection. In our experience, debridement, prolonged antibiotics, and two‐stage/three‐ stage prosthesis implantation could be considered as a treatment strategy for hip osteomyelitis involving *G.* a*diacens*.

## 1. Introduction

Osteomyelitis is generally caused by bacteria and is associated with a variable degree of bone loss. Bacteria may reach the bone through both exogenous (i.e., open fractures, surgery, and surgical wound infections) and endogenous (i.e, hematogenous, contiguous, or lymphatic) sources [1].

According to Cierny–Mader, osteomyelitis could be classified into four types: medullary (Type 1), superficial (Type 2), localized (Type 3), and diffuse (Type 4). While the patients can be grouped into 3 categories: patients in apparent good health (Group A), compromised patients (Group B), and patients too fragile for surgery (Group C) [2].

However, the diagnosis of osteomyelitis might be extremely difficult, particularly in the cases of chronic disease and/or low‐virulent pathogens.

After vertebral localization, the hip is among the most common sites for the development of osteomyelitis, mostly due to *Staphylococcus aureus* (especially methicillin‐resistant *Staphylococcus aureus* [MRSA] variants) [2].

*Granulicatella adiacens* (*G. adiacens*) is considered a normal commensal of the human mouth microbiota; although in some patients, it could be associated with endocarditis and bacteremia. Bone involvement in *G. adiacens*‐related infection is extremely rare. In fact, in the available literature, only 19 cases of *G. adiacens*‐related musculoskeletal infections were described, mostly associated with joint replacements [3–18].

Osteomyelitis of a long bone due to *G. adiacens* has not been described yet. In the present study, we present a CAse REports (CARE)–checklist‐compliant history of a patient with *G. adiacens*‐related hip osteomyelitis [19]. In the present paper, we focused on difficulties in diagnosis as well as pharmacological and surgical management. A brief commentary on the available evidence was also performed (the discussed papers are listed in Table 1).

**TABLE 1:** List of articles discussed in the multidisciplinary service of infectious diseases and orthopedics.

Author	Year	Journal	Number of cases	Diagnosis	Sex	Age	Surgery	Other surgery	Antibiotic therapy	Duration of the antibiotic therapy (weeks)	Infection outcome	Clinical outcome (functional result)	Follow‐up (months)
Quénard et al. [3]	2017	BMC Musculoskelet Disord.	1	Hip PJI	Male	75	Two‐stage prosthesis revision	No	Oral amoxicillin, oral clindamycin	24	Without infection	N.A.	24

Quénard et al. [3]	2017	BMC Musculoskelet Disord.	1	Hip PJI	Female	44	DAIR	No	Intravenous imipenem/cilastatin and oral ciprofloxacin^∗^; oral amoxicillin and oral ciprofloxacin^∗∗^	4; 20	Without infection	N.A.	16

Aweid et al. [13]	2016	JMM Case Reports	1	Hip PJI	Male	81	DAIR	DAIR	Vancomycin 500 mg, piperacillin/tazobactam 4.5 g and fusidic acid 500 mg^∗^ daptomycin 60 mg and meropenem 2g^∗∗^	1^∗^ 5^∗^		N.A.	N.A.

Badrick et al. [14]	2020	IDCases	1	Hip PJI	Male	79	Two‐stage revision	No	Benzyl penicillin	6	Without infection	Full recovery	60

Quenard et al. [3]	2017	BMC Musculoskelet Disord.	1	Knee PJI	Male	65	One‐stage revision	No	Oral rifampicin, oral clindamycin	24	Without infection	N.A.	24

Riede et al. [4]	2004	Scand J Infect Dis	1	Knee PJI	Male	43	Two‐stage prosthesis revision^∗^	No	Amoxicillin, amikacin (first stage); amoxicillin, amikacin, and rifampicin (second stage)	6; 2	Infection recurrence	Satisfying functional result	24

Mougari et al. [5]	2013	J Med Microbiol	1	Knee PJI	Male	55	Two‐stage prosthesis revision	No	Intravenous amoxicillin and rifampicin (first stage); oral amoxicillin and rifampicin (second stage)	2; 24	Without infection	N.A.	24

Pingili et al. [6]	2017	IDCases	1	Knee PJI	Male	64	Two‐stage prosthesis revision	No	Intravenous ertapenem^∗^; cefazolin^∗∗^	6^∗^; 3 days^∗∗^	Not specified	symptomatic	8

Lovering et al. [20]	2025	Case Rep Infect Dis	1	Knee PJI	Female	66	Two‐stage prosthesis revision	No	Intravenous vancomycin	Not specified	N.A.	N.A.	N.A.

Narayana Murthy et al. [16]	2021	Acupuncture in Medicine	1	Knee PJI	Male	65	Arthroscopy	Open debridement	i.v. vancomycin followed by clindamycin 450 mg^∗^	6	Asymptomatic	Fully mobile knee without pain	36

Hepburn et al. [12]	2003	Rheumatol In	1	Knee septic arthritis	Female	68	Arthroscopic irrigation^∗^	Arthroscopic irrigation^∗∗^	Clindamycin followed by cephalexin^∗^, cefazolin^∗∗^, gentamicin^∗∗∗^	4^∗^, 2^∗∗^	Without infection	Well	6

M.Roson et al. [15]	2018	Rev Esp Cir Ortop Traumatol.	1	Knee septic arthritis after LCA reconstruction	Male	40	Arthroscopic washing and debridement	No	i.v. amoxicillin/clavulanic switched with oral therapy	10 days i.v. and 6 weeks oral	Without infection	Returned to sport	24

Rosenthal et al. [8]	2002	Infection	1	Vertebral osteomyelitis	Male	68	No	No	Penicillin, rifampin, and gentamicin (after being discontinued because of resistance)		Without infection	Well, but residual back pain	6

Heath et al. [9]	1998	Aust N Z J Med	2	Vertebral osteomyelitis	Male	45	No	No	Intravenous penicillin and gentamicin (for endocarditis)^∗^, intravenous penicillin^∗∗^, oral clindamycin^∗∗∗^	4^∗^, 2^∗∗^, 2^∗∗∗^			
				Vertebral osteomyelitis	Male	50	No	No	Oral ciprofloxacin and fusidic acid (empirical)^∗^, intravenous penicillin and gentamicin^∗∗^, intravenous ceftriaxone^∗∗∗^	24^∗^, 2^∗∗^, 2 ^∗∗∗^			

Uehara et al. [10]	2013	Int J Surg Case Rep	1	Vertebral osteomyelitis	Female	48	No	No	Intravenous ampicillin	6	Without infection	Well	6

Fukuda et al. [11]	2010	Tokai J Exp Clin Med	1	Vertebral osteomyelitis	Male	73	No	No	Penicillin G and gentamicin^∗^, amoxicillin	4^∗^	Without infection		

Van Der Palen et al. [17]	2021	Case Reports in Infectious Diseases	1	Vertebral osteomyelitis	Male	45	No	No	i.v. penicillin followed by oral clindamycin (600 mg)	3; 2	Negative markers and blood cultures	Fully recovered	6 weeks

York et al. [18]	2016	Cureus Journal of Medical Science	1	Vertebral Osteomyelitis	Male	46	L2 vertebral body CT‐guided biopsy	Posterior decompression and L1‐L3 fixation L2 corpectomy and interbody cage placement	Vancomycin and ceftazidime^∗^; vancomycin^∗∗^	6	N.A.	N.A.	N.A.

Present study			1	Hip osteomyelitis	Male	59	Two‐stage implantation	Revision for hip dislocation	Doxycycline 100 mg and Cotrimoxazole 800 mg/160 mg	12	Without infection	OHS 29	24

tot	20		23		16 M; 7 F		Two stage 5; Irrigation 3	Two stage 1; Irrigation 1; Revision 1		15.7	13 without infection; infection recurrence		17,1

*Note:* In case of repeated surgeries/two‐stage surgeries, “^∗^” indicates the first surgery followed by the antibiotic therapy and “^∗∗^” stands for the second surgery and the second antibiotic therapy. M, male; F, female; tot, total.

Abbreviations: N.A., not available; OHS, Oxford Hip Score; PJI, periprosthetic joint infection.

## 2. Case Presentation

This article reports on the case of a 59‐year‐old patient with hip osteomyelitis receiving a regular clinical and radiological assessment at regular intervals of follow‐up in our multidisciplinary service of orthopedics and infectious diseases. The patient’s functional status was assessed using the Oxford Hip Score (OHS) [21]. Patients’ informed consent was obtained for anonymous data publication. According to the Italian law, a formal ethical approval was not required because the present study includes routinely performed clinical and radiological evaluations.

The patient had a history of absence‐type epilepsy associated with episodes of loss of consciousness resulting in falls, constant substance use disorder (cocaine and heroin) for 10 years before our observation, with some relapses during this time. No prior surgical procedures were reported.

The patient was admitted to our multidisciplinary Infectious Diseases and Orthopedic service in June 2022 on methadone therapy. He complained about persistent low back and groin pain. A swelling of the left lower limb was observed since October 2021.

**FIGURE 1:**
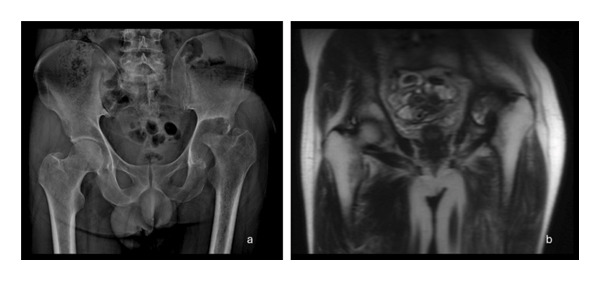
Preoperative X‐rays (a) and MRI (b). Note the amount of bone resorption on both the acetabulum and the femoral head.

The medical examination at the time of our first observation showed a painful and very restricted range of motion (ROM) of the left hip, with severe hypotonia of the left hamstring and quadriceps muscles. A lower limb discrepancy of 4 cm was also detected. No clinical signs of any spine alteration were observed at that time. The OHS collected at the first observation was 2/40. A comprehensive imaging and laboratory examination was then conducted, and both the X‐rays and the magnetic resonance imaging (MRI) showed signs of abundant bone resorption of the femoral head due to a bone infection (Figure 1). Laboratory tests improved the suspicion of an active infection (erythrocyte sedimentation rate [ESR]: 31 mm/h [range 2–15 mm/h], C‐reactive protein [CRP]: 3.6 mg/dL [range 0–1 mg/dL], and WBC: 8200/μL [range 4000–11000 × 10^^3^/μL]).

**FIGURE 2:**
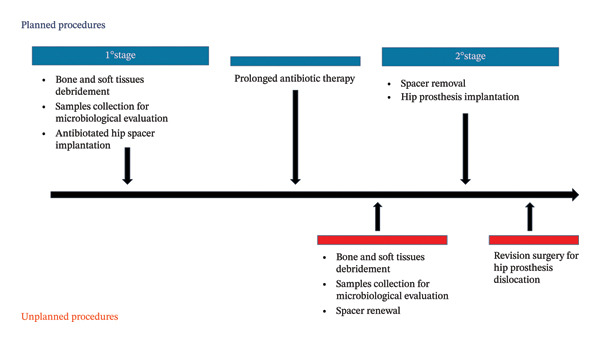
Timeline of planned (blue) and unplanned (red) surgical procedures in our patient.

**FIGURE 3:**
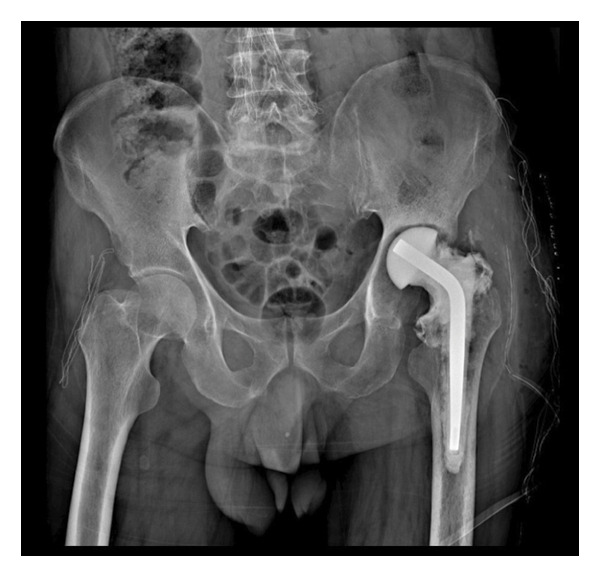
Postoperative X‐rays at the time of the first spacer implantation.

After the multidisciplinary evaluation of the case, a multistage surgical procedure was planned (see Figure 2 for further details). The first stage consisted of surgical debridement and implantation of an antibiotic‐cemented spacer (Spacer G, Tecres S.p.A., Sommacampagna [VR], Italy) (Figure 3). During this procedure, 8 samples for microbiological evaluation were also collected and resulted positive for both *Staphylococcus aureus* (meticillin‐resistant *S. aureus* [MRSA]) (1) and (3) *G. adiacens*). The *G. adiacens* was identified after multiple cultures on CAN Agar Blood in microareophilia and subsequent matrix‐assisted laser desorption/ionization time‐of‐flight (MALDI‐TOF). From pertinent cultures, a subsequent 16S rRNA gene sequencing was also performed to confirm the diagnosis. In order to clarify the possible sources of the *G. adiacens* hip osteomyelitis, during the hospitalization, the patient underwent a transthoracic echocardiogram that did not show signs of endocarditis. Moreover, although the oral care of the patient was poor, his history was negative for any oral procedures in the months before the onset of the clinical symptoms. As soon as the microbiological samples tested positive and the sensitivities became available, the patient started an oral antibiotic therapy based on the combination of doxycycline 100 mg bis in die (bid) and cotrimoxazole 800 mg/160 mg ter in die (tid) and was then discharged with the recommendation of toe‐touch weight‐bearing.

The patient was followed up at regular intervals both clinically and biochemically. Three months after the surgical debridement and spacer implantation, the patient was pain‐free, and the laboratory tests (CRP, ESR, and WBC count) were negative. However, a mild rubor and calor were still reported around the surgical approach. The MRI conducted at that time was inconclusive, and therefore, considering the high recurrence rate reported in the available literature, the patients underwent a newer surgical debridement and spacer renewal. Also, during this second procedure, samples for microbiological evaluation were collected. At this point, the microbiological tests resulted negative, and CRP was 2.42 mg/dL (range: 0–1 mg/dL). At discharge, the patient followed an antibiotic therapy with doxycycline 100 mg tid and sulfamethoxazole and trimethoprim 160 + 800 mg 2 vials tid.

**FIGURE 4:**
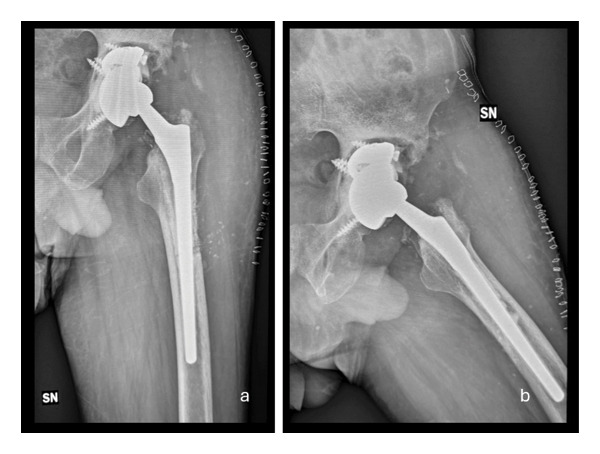
Anteroposterior (a) and axial (b) postoperative X‐rays at the time of the THA.

Three months after the second surgery, after a total of 6 months of antibiotic therapy (discontinued on 02/2023), both clinical and laboratory tests were negative for active infection, and therefore, the patient underwent a total hip arthroplasty (THA), using a revision shell with superior pole augmentation and a long cylindrical uncemented stem (Zimmer Biomet, Warsaw IN 46580) (Figure 4). During this phase, the intraoperative microbiological samples resulted again negative.

The patient was discharged with recommendations to avoid hip abduction and flexion.

**FIGURE 5:**
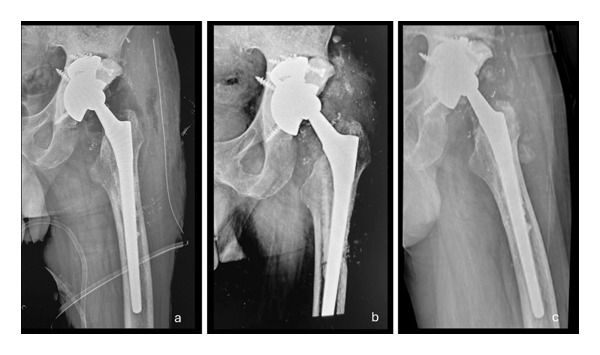
Anteroposterior postoperative X‐rays at the time of THA revision with a dual mobility cup (a). (b and c) Anteroposterior and axial X‐rays at 4 months after the revision surgery.

Unfortunately, 4 months after the THA, the prothesis dislocated because of an extreme abduction. A partial revision of the acetabulum was performed using a dual mobility cup (Figure 5). The intraoperative samples collected at this time point were reported to be negative again.

Four months after the last surgery, the prosthesis was clinically and radiographically stable, and there were no signs of active infection. However, the OHS slightly improved at the last follow‐up (17/40).

## 3. Discussion

### 3.1. Characteristics of the *G. adiacens* and Difficulties in Diagnosis

*G. adiacens* is a nutritionally variant *Streptococcus* (NVS) difficult to detect in standard blood culture, because its identification needs the addiction of pyridoxal or cysteine supplementation [22]. Therefore, it could be assumed that, for other NVS, *G. adiacens* might be an undiscovered cause of several culture‐negative infections.

NVS are part of the healthy oral, intestinal tract, and urogenital system microbiota [22]. Commonly, *G. adiacens* is observed in the oral cavity and in dental plaque, causing endodontic infections and dental abscesses [23].

*G. adiacens* was frequently associated with generalized infections with bacteremia and endocarditis, especially in patients with substance use disorder [8, 22, 24–28]. Less commonly, the *G. adiacens* had been observed in surgical‐related infections, such as postinstrumentation meningitis, breast implants, and peritoneal dialysis–related peritonitis [29].

In order to improve *G. adiacens* detection, some authors suggested the use of MALDI‐TOF, a technique used to identify and analyze large biological molecules such as proteins and DNA. The procedure consists of two different phases. The first is the “MALDI” part, where a laser is used to vaporize and ionize molecules embedded in a matrix. The second phase is the “TOF,” which separates ions based on their mass [30].

However, although MALDI‐TOF represents a rapid and increasingly available diagnostic tool, its accuracy in identifying NVS may be limited, particularly in distinguishing closely related species. In this context, molecular methods, especially 16S rRNA gene sequencing, are currently considered the gold standard for the identification of *G. adiacens*.

Indeed, 16S rRNA gene sequencing allows a highly accurate identification, overcoming the limitations related to its slow growth and specific nutritional requirements in culture. Particularly, Woo et al. demonstrated the utility of 16S rRNA gene sequencing in characterizing *Granulicatella* and *Abiotrophia* bacteremia, highlighting its crucial role in the detection of these difficult‐to‐culture pathogens [31].

Moreover, comparative studies have shown that while MALDI‐TOF MS is a valuable and rapid diagnostic technique, it may be less reliable than molecular approaches in certain clinical scenarios. Ratcliffe et al. reported discrepancies between MALDI‐TOF and conventional identification systems, suggesting that molecular confirmation may be required, particularly in invasive infections caused by NVS [32].

In our case, the use of 16S rRNA gene sequencing was essential to confirm the microbiological diagnosis.

### 3.2. Musculoskeletal Infection of *Granulicatella adiacens*

Musculoskeletal infection (including the implant‐related and those affecting both the bone and the joint) caused by *G. adiacens* is rarely reported. To the best of our knowledge, our case represents the twentieth case of musculoskeletal infection reported. A research on the available literature was conducted using the PubMed database by two independent reviewers (G.N. and D.L.I.). The following terms in their various combinations were used: “hip osteomyelitis,” “bone infection,” “*Granulicatella adiacens*,” and “septic arthritis.”

In detail, in the available literature, seven cases of vertebral osteomyelitis [8–11, 17, 18], two cases of knee arthritis [12, 15], and 10 cases of periprosthetic joint infection (PJI) [3–7, 13, 14, 16] had been described (see Table 1 for further details). However, *G. adiacens* might be underreported, especially in PJI, due to its slow growth characteristics and the need for specific techniques for its identification [33].

### 3.3. Diagnosis of Musculoskeletal Infection

In the *G*. *adiacens*‐related PJI, the diagnosis was based both on clinical signs (i.e., reddish and painful hip/knee prosthesis, fistula and purulent drainage from the prosthesis surgical scar, and eventually fever or cyst formation) and investigation (laboratory tests, i.e., CRP, ESR and a leukocyte count, and radiological exams) [3]. In all cases, intraoperative samples and synovial fluid microbiological cultures confirmed the diagnosis of *G*. *adiacens* using the MALDI‐TOF mass spectrometry or 16S rRNA gene sequencing. Considering that *G. adiacens* is part of the mouth microbiota, an accurate evaluation of the patient’s dental well‐being is mandatory. Among the patients with *G. adiacens*‐related PJI, three patients underwent oral hygiene procedures few months before the onset the symptoms, without proper antibiotic prophylaxis [3, 5, 6, 13, 14].

### 3.4. Treatment of Musculoskeletal Infection of *G. adiacens*

As a common law, most of the musculoskeletal infections should undergo a multidisciplinary approach based on surgical debridement and antibiotic therapy. Hip osteomyelitis may be extremely difficult to treat (DDT), considering the need for extended bone debridement that may lead to severe bone loss and difficulties in joint reconstruction [34].

Very often, hip osteomyelitis is treated through the transient application of a cemented spacer, such as in PJI [3]. In our opinion, the most difficult evaluation for the treatment of musculoskeletal infections is the identification of the appropriate timing for surgery and antibiotic therapy.

This is particularly evident in rare cases in which no clear recommendations are available, such as *G. adiacens*‐related infections.

### 3.5. Surgical Treatments

*G. adiacens*‐related PJI mostly needed to undergo multiple surgical procedures and prolonged antibiotic therapy [3]. Generally, the treatment of PJI could be based on debridement and implant retention (DAIR), one‐stage revision or two‐stage revision, each with its indications and limits [35–37]. Among the 10 cases of *G. adiacens*‐related PJI, three cases were treated with DAIR, one case with one‐stage revision, and six cases were treated with two‐stage revision.

DAIR is associated with high rates of infection recurrence [38]. However, its reliability could be high when the following criteria are met: (1) the prosthesis is stable, (2) a pathogen with susceptibility to antimicrobial agents is active against surface‐adhering microorganisms, (3) there is no sinus tract or compromised soft tissue, and (4) the symptom duration of infection is less than 3 weeks [39]. Moreover if the pathogen is susceptible to rifampin (i.e., gram‐positive pathogens) or ciprofloxacin (for gram‐negative pathogens), the rate of effectiveness could rise up to 90% [40]. Moreover, recently, the addition of local antibiotic delivery means seemed to further reduce the infection recurrence rate of PJI treated with DAIR [41]. DAIR resulted effective in the treatment of one *G. adiacens*‐related PJI, considering that the patient subsequently did not undergo implant removal [3].

One‐stage revision consists of implant removal, debridement, and reimplantation of a new prosthesis [35]. Obviously, an adequate antibiotic therapy is always required. One‐stage revision is generally indicated in patients with good bone stock and no‐sinus tract, preoperative identification of a non‐DTT bacterium (infections caused by pathogens resistant to biofilm‐active antimicrobials) [42]. Although the 2‐stage approach has become the method of choice for most surgeons, a single center–based experience on PJI treated with one‐stage revision reported a success rate of over 85% [43]. One case of *G. adiacens*‐related knee PJI described in the literature was treated with one‐stage revision [3]. During the 6 months after the implant revision, the patient was treated with oral rifampicin, 300 mg, tid, and oral clindamycin, 9 g, tid. No relapse was observed during the 2‐year postantimicrobial follow‐up [3].

Two‐stage revision means the application of a temporary spacer after the removal of the infected prosthesis, and the subsequent re‐implantation of a new arthroplasty after the infection was controlled with the use of antibiotics [35]. Two‐stage revision is considered the gold standard for the treatment of PJI, with a success rate of up to 90% [36, 44, 45].

However, the time between the first and the second stage is variable [44]. The approach of short intervals (2–4 weeks) might be suitable for patients with an easy‐to‐treat bacterium and mildly compromised soft tissue, whereas in other cases, at least 2 months of antibiotic therapy are generally needed [44].

A two‐stage revision was performed by Quenard et al. with G. *adiacens-*related PJI. The second stage was performed after 3 months, albeit with the occurrence of spacer dislocation. A prolonged antibiotic therapy was anyway used (6 months of oral amoxicillin, 2 g, tid; oral clindamycin, 9 g tid). No infection relapse was observed during the 2‐year of follow‐up [3].

Another case of two‐stage prosthesis revision was described by Mougari et al. [5]. The second stage was performed 4 months after the spacer implantation. During the interval between the two stages, the patient received prolonged antimicrobial therapy with amoxicillin (2 g three times daily) in combination with rifampicin (600 mg three times daily). In the 2 years of subsequent follow‐up, no signs of infection recurrence were observed [5].

Badrick et al. also reported a two‐stage revision for a THA PJI caused by *G. adiacens*. The second stage was performed 10 weeks after the first surgery and after a core biopsy showing no signs of infection. During the interim period, intravenous benzylpenicillin was administered for 6 weeks [14].

Two cases of *G. adiacens*‐related knee septic arthritis were also described. The first in a 68‐year‐old female [12]. The patient underwent arthrocentesis and arthroscopic irrigation. The clinical symptoms (knee pain and fever) improved after the procedure. However, 2–3 months later, she reported the gradual re‐onset of left knee swelling and pain. This time, the *G. adiacens* was found in the synovial fluid aspirated. She started antibiotic therapy based on cefazolin at 2 g tid, with gentamicin (70 mg bid) for 2–4 weeks. During this period, the patient underwent several arthroscopic irrigations of her knee till the synovial fluid cultures were reported to be negative. No evidence of infection recurrence was observed in the 6‐month of follow‐up [12].

The second case of septic arthritis in a 40‐year‐old male [15] occurred 10 days after an ACL reconstruction, with acute pain and swelling of the knee, which prevented the patient from sleeping. The patient had no fever, and the knee was cold and edematous. *G. adiacens* was isolated in synovial fluid obtained by arthrocentesis. The patient underwent arthroscopic washing and debridement, followed by 10 days of intravenous antibiotic treatment with amoxicillin/clavulanic acid 2 g every 8 h, switched to oral therapy for an additional 6 weeks. After a 24 months follow‐up, the patient showed no sign of recurrent infection; the knee was stable with a ROM of 0°–130° of flexion, and the patient had returned to sport without pain or instability [15].

Although in all cases, good infection control was reported, in none of them, the joint function and/or the patient quality of life was evaluated using a validated tool. Therefore, it is difficult to provide a definitive conclusion on the outcomes of *G. adiacens* musculoskeletal infections surgically treated. In our patient, the joint function was poor at the final follow‐up. However, the occurrence of bone infection, including PJI, severely affects patients’ quality of life [46].

### 3.6. Antibiotic Treatment

There is no consensus on the best available therapy and the ideal duration of treatment in *G. adiacens* musculoskeletal infections [4].

*G. adiacens* is often susceptible to different classes of antibiotics with activity against gram‐positive bacteria. Regarding treatment, according to AHA guidelines, in cases of endocarditis or other severe infections caused by *Granulicatella spp*, the therapy of choice is penicillin or ceftriaxone [47]. However, it is crucial, especially in the case of musculoskeletal infections, to evaluate the choice of antimicrobial therapy based also on the drug’s ability to penetrate the bone.

Most of the cases available in literature were published before the Oral versus Intravenous Antibiotics for Bone and Joint Infection (OVIVA) trial [48] on the noninferiority of oral over intravenous administration of antibiotics was conducted. Therefore, they received intravenous drugs more often for the entire therapeutic course or for a significant portion of the treatment [49].

In the case series of Quenard et al., three cases of infection caused by *G. adiacens spp* were described [3]. In two cases, hip was involved: in one case, after 3 months of antibiotic therapy with ciprofloxacin and fusidic acid, it was performed based on DAIR followed by 1 year of treatment with oral rifampicin and oral ofloxacin; in the other one, the infection was treated with DAIR and antimicrobial treatment, first imipenem/cilastatin in combination with oral ciprofloxacin, and then 5 months of oral therapy with ciprofloxacin and amoxicillin. Eventually, the patient underwent a two‐stage reprosthetic surgery followed by 6 months of antibiotic therapy with amoxicillin and clindamycin. In the last cases presented in the series, the knee was involved and treated initially with DAIR followed by two‐stage exchange arthroplasty in association with 8 months of oral ciprofloxacin and ofloxacin [3]. No relapse was observed in any of the cases they presented.

Two other cases were about periprosthetic infection of the hip. In Aweid et al.’s case report, the patient underwent an unsuccessful DAIR for a supposed G. *adiacens.* Several antibiotic treatments were applied with a positive biochemical response after daptomycin (60 mg once daily) and meropenem (2 g every 8 h) IV antibiotics. Unfortunately, the patient died before the two‐stage revision protocol started [13].

In Badrick et al.’s case, the patient underwent a two‐stage revision surgery, followed by treatment with benzyl penicillin administered by continuous infusion for 6 weeks. Two weeks after completing the therapy, a core biopsy showed no signs of ongoing infection, and the patient underwent second‐stage revision [14].

Knee involvement was also described in Riede, Mougari, and Narayana Murthy’s case reports: in the first one, a DAIR was performed, followed by recurrence of infection; thus, the patient was treated with a two‐stage prosthesis reimplant and 8 months of antimicrobial therapy with rifampicin and ofloxacin [4, 5]. After a second microbiological and clinical failure, the patient underwent a one‐stage revision and 6 months of rifampicin and clindamycin [4].

In Mougari et al.’s case report, after removal of the knee prosthesis, a gentamicin‐loaded cement spacer was implanted, and the patient started an intravenous treatment with amoxicillin and amikacin; after a 6‐week course of therapy and an antibiotic holiday of 2 weeks, reimplantation of a total knee arthroplasty was followed by 2 weeks of postoperative treatment with amoxicillin, amikacin, and oral rifampicin [5].

Regarding the case reported by Narayana Murthy et al., after a diagnostic arthroscopy used to collect biopsy samples, the patient started intravenous vancomycin followed by intravenous clindamycin 450 mg four times daily for 6 weeks. During this period, the patient also underwent an open debridement to reduce bacterial load. At the final follow‐up, the patient was infection‐free [16].

Two cases of septic arthritis were found, both involving the knee and treated through arthroscopic irrigation and general antibiotics. In one case, the patient received clindamycin during the perioperative period, and then cephalexin upon discharge. After 2‐3 months, a gradual recurrence of symptoms was reported, so the patient received an intravenous course of antibiotic treatment with cefazolin in combination with gentamicin for 4 and 2 weeks, respectively, and underwent a second arthroscopic irrigation [12].

In the second, the patient started 10 days of i.v. antibiotic treatment with amoxicillin/clavulanic acid tid followed by oral administration of the same antibiotic for an additional 6 weeks [15].

Our patient received a total of 5 months of doxycycline 100 mg tid and sulfamethoxazole and trimethoprim 2 vials tid, reporting a reliable infection control.

Current evidence appears to support the use of prolonged antibiotic therapy for *G. adiacens*‐related infections, with treatment durations of up to 6 months, similar to the approach adopted for difficult‐to‐treat (DTT) bone infections.

### 3.7. *G. adiacens*‐Related Vertebral Osteomyelitis

Seven cases of vertebral osteomyelitis have been described [8–11, 17, 18]. All but one patient with *G. adiacens*‐related vertebral osteomyelitis complained of back pain and fever [8–11, 17] [18]. All patients underwent MRI and/or computed tomography (CT) scans, which revealed imaging features compatible with osteomyelitis, such as increased radiolucency of the intervertebral disc and the adjacent vertebrae [8–11, 17, 18]. In these cases, the CT‐guided biopsy might be particularly useful to collect samples of the intervertebral discs and complete the diagnosis. All but one of the cases of *G. adiacens*‐related vertebral osteomyelitis were conservatively treated [8–11, 17]. One case of L2 vertebral osteomyelitis was treated through L1–L3 decompression and fixation and a L2 corpectomy with an interbody cage placement. In this case, antibiotic therapy started after the diagnostic CT‐guided biopsy performed before surgery and continued postoperatively [18].

Regarding antibiotic therapy, all cases examined, except for one managed with combined surgical and antibiotic treatment [18], were treated conservatively with antimicrobial therapy alone [8–11, 17]. The antibiotic regimens mainly consisted of combinations of aminopenicillins or cephalosporins with aminoglycosides [8–11, 17].

## 4. Conclusion

*G. adiacens* is a virulent pathogen DDT in cases of bone infection. A correct diagnosis is crucial and could be obtained by prolonged cultures of multiple surgical biopsies and/or specific identification techniques such as molecular tools or MALDI‐TOF. In our case, hip osteomyelitis may have been facilitated by the substance use disorder, which could increase his susceptibility to unexpected infections. Our case report, along with the others reported in the available literature, underlined the need for multiple surgeries followed by prolonged antibiotic treatment to control the infection. Little is known about the clinical outcomes of *G. adiacens*‐related musculoskeletal infection. However, for other bone infections, it negatively affects the patients’ quality of life and joint function. We believe that the present study could be of aid as a guide for further similar cases.

## Funding

No funding was obtained.

## Conflicts of Interest

The authors declare no conflicts of interest.

## Supporting Information

Additional supporting information can be found online in the Supporting Information section.

## Supporting information


**Supporting Information** CARE‐checklist‐*Granulicatella*.

## Data Availability

The data that support the findings of this study are available from the corresponding author upon reasonable request.
