# Supplementary material for: Osteomyelitis of the Hip due to Granulicatella adiacens: A Case Report of an Extremely Rare Cause of Bone Infection
Source: Case Rep Infect Dis. 2026 Jul 22;2026:7967434. doi: 10.1155/crdi/7967434 (PMC13392416; doi:10.1155/crdi/7967434)
Supplement: Supplementary file 1 — Supporting Information CARE‐checklist‐Granulicatella. [file CRDI-2026-7967434-s001.pdf]

CARE case report guidelines

CARE Checklist of information to include when writing a case report

(cc) BY-NC-ND

Topic	Item	Checklist item description	Reported on Line
Title	1	The diagnosis or intervention of primary focus followed by the words "case report"	1-3
Key Words	2	2 to 5 key words that identify diagnoses or interventions in this case report, including "case report"	60-61
Abstract (no references)	3a	Introduction: What is unique about this case and what does it add to the scientific literature?	32-38
	3b	Main symptoms and/or important clinical findings	41-43
	3c	The main diagnoses, therapeutic interventions, and outcomes	44-52
	3d	Conclusion—What is the main "take-away" lesson(s) from this case?	55-59
Introduction	4	One or two paragraphs summarizing why this case is unique (**may include references**)	85-110
Patient Information	5a	De-identified patient specific information.	114-121
	5b	Primary concerns and symptoms of the patient.	127-128
	5c	Medical, family, and psycho-social history including relevant genetic information	122-124
	5d	Relevant past interventions with outcomes	124-125
Clinical Findings	6	Describe significant physical examination (PE) and important clinical findings.	129-132
Timeline	7	Historical and current information from this episode of care organized as a timeline	122-199
Diagnostic Assessment	8a	Diagnostic testing (such as PE, laboratory testing, imaging, surveys).	133-139
	8b	Diagnostic challenges (such as access to testing, financial, or cultural)	139-141
	8c	Diagnosis (including other diagnoses considered)	142-154
	8d	Prognosis (such as staging in oncology) where applicable	
Therapeutic Intervention	9a	Types of therapeutic intervention (such as pharmacologic, surgical, preventive, self-care)	136-141 169-173
	9b	Administration of therapeutic intervention (such as dosage, strength, duration)	152-156
	9c	Changes in therapeutic intervention (with rationale)	178-180
Follow-up and Outcomes	10a	Clinician and patient-assessed outcomes (if available)	117
	10b	Important follow-up diagnostic and other test results	192-194
	10c	Intervention adherence and tolerability (How was this assessed?)	165
	10d	Adverse and unanticipated events	188-190
Discussion	11a	A scientific discussion of the strengths AND limitations associated with this case report	197-452
	11b	Discussion of the relevant medical literature **with references**.	197-452
	11c	The scientific rationale for any conclusions (including assessment of possible causes)	458-460
	11d	The primary "take-away" lessons of this case report (without references) in a one paragraph conclusion	455-466
Patient Perspective	12	The patient should share their perspective in one to two paragraphs on the treatment(s) they received	196-197
Informed Consent	13	Did the patient give informed consent? Please provide if requested	Yes ☒ No ☐
